# Consensus on Human Epidermal Growth Factor Receptor 2 Overexpression Testing in Pan‐Tumor

**DOI:** 10.1002/cai2.70060

**Published:** 2026-04-21

**Authors:** Zhe Wang, Lin Chen, Wei Rao, Baoshan Cao, Muyan Cai, Xiangshan Fan, Yuan Ji, Yuan Li, Yan Song, Yan Sun, Yu Sun, Chunyan Wu, Qingxin Xia, Yanfeng Xi, Liyan Xue, Rutie Yin, Han Liang, Jianming Ying

**Affiliations:** ^1^ Department of Pathology, Xijing Hospital and School of Basic Medicine Fourth Military Medical University Xi'an China; ^2^ Department of Gastrointestinal Surgery Peking University International Hospital Beijing China; ^3^ Department of Pathology, National Cancer Center/National Clinical Research Center for Cancer/Cancer Hospital Chinese Academy of Medical Sciences & Peking Union Medical College Beijing China; ^4^ Department of Medical Oncology and Radiation Sickness Peking University Third Hospital Beijing China; ^5^ Department of Pathology, Sun Yat‐sen University Cancer Center, State Key Laboratory of Oncology in South China Guangdong Provincial Clinical Research Center for Cancer Guangzhou China; ^6^ Department of Pathology The First Affiliated Hospital of Anhui Medical University Hefei China; ^7^ Department of Pathology, Zhongshan Hospital Fudan University Shanghai China; ^8^ Department of Pathology Fudan University Shanghai Cancer Center Shanghai China; ^9^ Department of Pathology Tianjin Medical University Cancer Institute & Hospital Tianjin China; ^10^ Department of Pathology Peking University Cancer Hospital & Institute Beijing China; ^11^ Department of Pathology, Shanghai Pulmonary Hospital, School of Medicine Tongji University Shanghai China; ^12^ Department of Pathology Henan Cancer Hospital/Affiliated Cancer Hospital of Zhengzhou University Zhengzhou China; ^13^ Department of Pathology Shanxi Province Cancer Hospital Taiyuan China; ^14^ Department of Obstetrics and Gynecology, West China Second University Hospital Sichuan University Chengdu China; ^15^ Department of Gastric Surgery Tianjin Medical University Cancer Institute & Hospital Tianjin China

**Keywords:** consensus, human epidermal growth factor receptor 2, overexpression, pan‐tumor, testing

## Abstract

Human epidermal growth factor receptor 2 (HER2) is a key biomarker and therapeutic target in several malignancies, including breast, gastric, and other solid tumors. Recent advancements in cancer molecular profiling and the Food and Drug Administration's approval of trastuzumab deruxtecan for HER2‐positive pan‐tumor indications have highlighted the broader relevance of HER2 alterations across diverse cancers. However, the lack of standardized guidelines for HER2 testing in a pan‐tumor context creates variability in clinical practice, hindering the optimal implementation of HER2‐targeted therapies beyond traditional indications. To address this gap, a multidisciplinary panel of Chinese experts has developed a consensus providing comprehensive recommendations on diagnostic strategies, testing methodologies, and clinical applications of HER2 overexpression detection. By establishing a unified framework for HER2 overexpression assessment, this consensus aims to enhance the precision of HER2 testing, optimize patient selection for targeted therapies, and improve clinical outcomes across a wide spectrum of HER2 overexpression malignancies.

## Graphical abstract

This is the first pan‐tumor human epidermal growth factor receptor 2 (HER2) testing consensus established by a national expert panel in China, addressing critical gaps in HER2 overexpression testing across solid tumors beyond breast and gastric cancers. This consensus marks a significant advancement in standardizing HER2 assessment for broader oncologic applications.

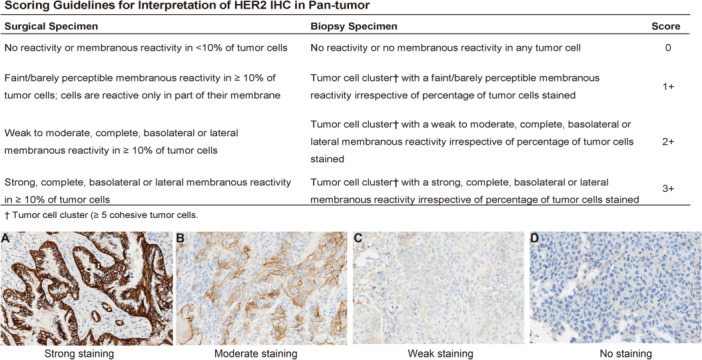

**Abbreviations**
CRC: colorectal carcinoma
DOR: duration of response
DV: disitamab vedotin
FDA: Food and Drug Administration
GEA: gastroesophageal adenocarcinoma
HER2: human epidermal growth factor receptor 2
NMPA: National Medical Products Administration
NSCLC: non‑small‑cell lung cancer
ORR: objective response rate
OS: overall survival
PFS: progression‐free survival
T‐DXd: trastuzumab deruxtecan

## 1 Background

Human epidermal growth factor receptor 2 (*HER2*), a member of the ErbB family of receptor tyrosine kinases, is crucial for regulating cellular processes, such as proliferation, differentiation, and survival [1]. Aberrant *HER2* signaling, primarily due to gene amplification or protein overexpression, contributes to the development and progression of various malignancies, including breast, gastric, ovarian, bladder, lung, and colorectal cancers. HER2 overexpression, which is associated with aggressive tumor behavior, is now a key target in precision oncology [2].

The clinical relevance of HER2 was first established with the advent of trastuzumab, which transformed the treatment landscape for HER2‐positive breast cancer [3, 4]. Subsequent research has expanded the use of HER2‐targeted therapies to other tumor types, notably gastric cancer, with demonstrated survival benefits. As a result, HER2 has evolved into a predictive biomarker across multiple malignancies [5, 6, 7, 8, 9]. However, despite encouraging results from trials, such as DESTINY‐PanTumor02, DESTINY‐Lung01, and DESTINY‐CRC02, the broader application of HER2‐targeted therapies remains limited [10, 11, 12]. This is largely due to the absence of standardized HER2 testing protocols beyond breast and gastric cancers, along with inconsistencies in diagnostic methodologies and scoring systems across tumor types. Current guidelines predominantly address breast and gastric cancers, leaving a gap in clinical guidance for other HER2‐expressing malignancies [13, 14].

To address this unmet need, a multidisciplinary panel of Chinese experts has developed a consensus on HER2 overexpression testing across diverse solid tumors excluding breast cancer and gastroesophageal adenocarcinoma (GEA). This document outlines recommendations on diagnostic strategies, testing methodologies, and clinical applications. By establishing a standardized framework for pan‐tumor HER2 overexpression testing, the consensus aims to improve patient selection, facilitate broader use of HER2‐targeted therapies, and ultimately enhance outcomes in patients with HER2‐overexpressing cancers.

## 2 Biological Significance of HER2 Overexpression

HER2, a member of the ErbB receptor family, is a key regulator of cellular growth and survival. Structurally, it consists of an extracellular ligand‐binding domain, a transmembrane segment, and an intracellular tyrosine kinase domain [15]. Unlike other family members, HER2 exists in a constitutively active conformation and does not require a specific ligand for activation; it readily forms homodimers or heterodimers (most potently with HER3), which trigger autophosphorylation and activate critical downstream signaling pathways, such as PI3K/AKT/mTOR [16, 17, 18, 19]. Under physiological conditions, HER2‐mediated dimerization and signaling are tightly regulated. Overexpression disrupts this balance, leading to amplified signaling and accelerated degradation of the cell‐cycle inhibitor p27^Kip1^, thereby driving uncontrolled cell proliferation, survival, and metastasis [20]. Given its central role in driving tumorigenesis and its structural accessibility, HER2 represents an ideal therapeutic target, with targeted therapies specifically designed to interrupt its oncogenic signaling.

## 3 Clinical Implications of HER2 Overexpression

### 3.1 HER2 Alterations Across Different Tumor Types

HER2 is implicated in the pathogenesis of 15%–20% of breast cancers [21], 15%–20% of GEAs [22], 8%–66% of ovarian cancers [23], 17%–80% of endometrial cancers [23], 6%–17% of bladder cancers [24], 2%–4% of lung cancers [25], 3%–5% of colon cancers [26], and 50% of head and neck cancers [27]. The biology of HER2 alterations varies by tumor type. Before HER2‐targeted therapies, *HER2* amplification was linked to poor prognosis, including higher recurrence, shorter survival, and brain metastases [28, 29, 30, 31, 32, 33]. In gastric and gastroesophageal junction cancers, HER2 is an established predictive biomarker for trastuzumab, as shown in the ToGA trial [34], though its prognostic value remains inconsistent [35, 36, 37, 38, 39, 40]. In non‐small‐cell lung cancer (NSCLC), HER2 protein overexpression and *ERBB2* amplification are weakly correlated and often result from chromosome 17 polysomy rather than focal amplification [41, 42]. In colorectal cancer, HER2 amplification/overexpression may drive resistance to anti‐EGFR therapies by sustaining ERK signaling, serving as a negative predictive biomarker [43]. In epithelial ovarian cancer, HER2 overexpression is associated with poorer survival and reduced treatment response, with variable significance across subtypes [1]. In endometrial cancer, especially uterine serous carcinoma, *HER2* amplification correlates with worse outcomes, with FISH‐positive cases showing particularly poor survival [44].

### 3.2 Clinical Evidence Supporting HER2 Overexpression as a Predictive Biomarker

Since the introduction of trastuzumab, HER2‐targeted therapies have markedly improved outcomes across multiple cancer types. Trastuzumab deruxtecan (T‐DXd) demonstrated substantial efficacy in several pivotal trials. In DESTINY‐PanTumor02, the objective response rate (ORR) was 37.1%, with a median duration of response (mDOR) of 11.3 months, progression‐free survival (PFS) of 6.9 months, and overall survival (OS) of 13.4 months in 267 patients with HER2‐expressing (immunohistochemistry [IHC] 3+/2+) solid tumors [10]. For HER2‐overexpressing nonsquamous NSCLC (DESTINY‐Lung01), confirmed ORRs were 26.5% (T‐DXd 6.4 mg/kg) and 34.1% (5.4 mg/kg) [11]. In HER2‐positive (IHC 3+ or IHC 2+/ISH+) metastatic colorectal cancer (mCRC) (DESTINY‐CRC02), confirmed ORRs were 37.8% (5.4 mg/kg) and 27.5% (6.4 mg/kg) [12]. Other HER2‐targeted agents also show activity. Pooled analysis of RC48‐C005 and RC48‐C009 trials demonstrated that disitamab vedotin (DV) yielded an overall confirmed ORR of 50.5%, median PFS of 5.9 months, and median OS of 14.2 months in pretreated HER2‐positive (IHC 3+/2+) locally advanced or metastatic urothelial carcinoma patients [45]. Furthermore, meaningful clinical responses to DV were also observed in other HER2‐positive solid tumors, such as salivary gland cancer, cholangiocarcinoma, female genital cancer, and extramammary Paget disease [46, 47, 48]. The ACE‐PanTumor01 found ARX788 had acceptable safety and antitumor activity in HER2‐positive advanced solid tumors [49, 50]. Preliminary data from HLX22‐001 showed a disease control rate of 36.4% and a median PFS of 44.0 days for HLX22 in patients with HER2‐positive solid tumors [51]. These results collectively reinforced HER2 overexpression as a viable pan‐tumor therapeutic target. On the basis of evidence from DESTINY‐PanTumor02, DESTINY‐Lung01, and DESTINY‐CRC02, the U.S. Food and Drug Administration (FDA) granted histology‐independent approval of T‐DXd for adult patients with unresectable or metastatic HER2‐positive (IHC 3+) solid tumors who have received prior systemic treatment and have no satisfactory alternative treatment options.

## 4 Methodology

This pan‐tumor HER2 testing consensus was developed through a systematic literature review, aimed at identifying relevant evidence from PubMed, Ovid, ASCO, and ESMO spanning the period from December 1, 2015, to December 2024. The review incorporated both MeSH terms and keywords related to pan‐tumor HER2, targeted therapies, and laboratory testing methodologies. Additional searches included clinical trial databases and authoritative websites. Studies were included if they were human‐based and addressed key questions related to laboratory testing methodologies for HER2, providing measurable data like predictive values, concordance, sensitivity, and specificity. Exclusion criteria encompassed non‐peer‐reviewed articles, animal studies, and those not involving tumors or relevant testing data. The quality of the studies was assessed based on study design, strength of evidence, and potential biases. All recommendations are accompanied by a level of evidence based on the Infectious Diseases Society of America‐United States Public Health Service Grading System (Table S1) [52], ensuring that the recommendations are robust and clinically applicable.

The consensus was formulated through a modified Delphi process involving a multidisciplinary expert panel composed of specialists from the departments of pathology, gastrointestinal surgery, oncology, and obstetrics and gynecology. The panel included experts with complementary expertise in clinical oncology and diagnostic pathology to ensure balanced perspectives. The development process consisted of three iterative Delphi rounds. In the first round, panel members independently reviewed the evidence summaries and provided structured feedback on draft statements covering HER2 testing scope, methodology, and interpretation standards. In the second round, all responses were aggregated and analyzed to identify statements lacking consensus, followed by revision and re‐evaluation through group discussion moderated by the consensus chairs. In the third round, a final vote was conducted to confirm endorsement of each recommendation, and statements achieving at least 80% agreement among panelists were adopted as final recommendations. This iterative, evidence‐based approach ensured objectivity, transparency, and broad expert agreement in formulating the pan‐tumor HER2 testing consensus.

## 5 Consensus Recommendations

### 5.1 Detection Necessity

HER2 is a key biomarker implicated in the development of various cancers [1], and its positivity is strongly associated with aggressive tumor behavior and poor prognosis [3, 4]. In the recent DESTINY‐PanTumor02, DESTINY‐Lung01, and DESTINY‐CRC02 studies, which played a pivotal role in the histology‐agnostic FDA approval of T‐DXd for HER2‐positive solid tumors, HER2 overexpression was defined across all eligible tumor types using the HER2 IHC criteria for GEA established by the ASCO/CAP [10]. In light of these advancements, there is growing recognition that HER2 testing should extend beyond breast and gastric cancers to encompass a broader range of tumor types. As HER2 positivity is associated with potential therapeutic benefits across various cancers, accurate detection of HER2 in a pan‐tumor context is crucial. This ensures that more patients can access targeted therapies, ultimately leading to improved clinical outcomes and prognoses.

### 5.2 Applicable Populations and Detection Timing

HER2 testing is essential for any patient being considered for HER2‐targeted therapy. Currently, the primary indications for HER2 testing include breast cancer, gastric cancer, and mCRC. Beyond these established indications, clinical trials continue to explore its role in lung, ovarian, bladder, biliary, and other cancers. As evidence evolves, the application of HER2 testing will continue to expand, solidifying its role as a cornerstone of precision oncology. Furthermore, due to spatiotemporal heterogeneity in HER2 expression, repeat HER2 testing should be performed on recurrent or metastatic tumor tissues whenever possible. This rebiopsy allows for a more accurate evaluation of HER2 status, reflecting the current state of the disease and accounting for potential changes in HER2 expression that may have occurred during disease progression.

*Recommendation* 1: HER2 testing should be performed for all pan‐tumor patients considered for HER2‐targeted therapy. For recurrent or metastatic lesions, tumor tissue should be obtained whenever possible for HER2 testing (evidence quality, Ⅱ; strength of recommendation, A).

### 5.3 Detection Methods

IHC is recommended as the standard method for detecting HER2 expression in pan‐tumor settings. Current HER2 IHC testing employs diverse primary antibody clones and staining platforms across clinical research and pathology labs. However, inconsistency in these choices between centers and trials hinders cross‐study comparisons and complicates establishing HER2 as a reliable, histology‐agnostic predictive biomarker. Common HER2 antibody clones (e.g., 4B5, HercepTest, CB11, SP3, A0485, 29D8, and MXR001) vary in epitope specificity, staining intensity, and background performance. When run on different automated platforms—each with distinct antigen retrieval, detection systems, and protocols—this reagent and workflow diversity contribute to analytical variability. Besides, variability in membrane staining completeness, intensity, or interpretability may lead to inconsistent scoring among laboratories and pathologists. Such discrepancies can affect patient selection for HER2‐targeted therapies and are especially concerning in pan‐tumor contexts, where HER2 expression patterns vary across tumor types, and validated scoring criteria are often lacking.

To minimize variability and improve reproducibility, it is critical to use HER2 testing assays that are approved by regulatory authorities, such as the FDA (US), National Medical Products Administration (China), and CE (Europe). These approvals ensure that test kits meet rigorous standards for accuracy, reliability, and safety. Recognizing that variations may exist between different HER2 testing kits, a consistency evaluation is essential to ensure comparability of results across various kits. This evaluation enhances the robustness of the testing process and ensures that healthcare professionals can trust consistent and accurate outcomes, irrespective of the kit used.

*Recommendation* 2: HER2 protein expression should be assessed using IHC, and validated platforms and certified antibodies are recommended to ensure accurate and reliable results (evidence quality, I; strength of recommendation, A).

### 5.4 Sample Types and Processing

Tumor tissue samples obtained through surgical resection or biopsy are the preferred specimen types for HER2 IHC testing. For patients from whom tissue samples cannot be obtained, cytological samples may serve as a potentially effective alternative. However, due to the difficulty in distinguishing between in situ and invasive tumors when using cytological samples, caution should be exercised when applying them, particularly to primary tumor sites. This limitation should be clearly documented in the pathology report.

All specimens should be fixed as soon as possible after removal (ideally within 30 min) and immersed in an adequate volume of 10% neutral buffered formalin. The optimal fixation time is 6–48 h, and should not exceed 72 h. Studies have shown that HER2 expression decreases gradually as the storage time of unstained slides increases [53]. To prevent antigen loss, unstained slides should be stored at room temperature for no longer than 6 weeks. Slides that have completed HER2 testing can be preserved long‐term using standard preservation methods. All HER2 testing should be accompanied by routine hematoxylin and eosin‐stained slides for comparison, ensuring an additional layer of context and accuracy in the evaluation process.

*Recommendation* 3: Tumor tissue samples are recommended for HER2 testing. A standardized specimen processing procedure should be established. All specimens should be promptly and adequately fixed after excision, with 10% neutral buffered formalin and a fixation time of 6–48 h being optimal. Unstained slides should not be stored at room temperature for more than 6 weeks (evidence quality, I; strength of recommendation, A).

### 5.5 Result Interpretation

Variation in HER2 IHC scoring criteria across different tumor types continues to challenge standardization. Various HER2‐targeted therapies are now widely used in clinical practice (Table 1); however, HER2 IHC scoring methods still vary considerably according to tumor type, clinical trial design, and the characteristics of the therapeutic agent. Despite ongoing efforts to define tumor‐specific criteria [54, 55, 56], universally accepted HER2 IHC scoring standards exist only for breast and gastric cancers. The variability in scoring criteria complicates standardized pathology reporting. A simple binary classification, for example, labeling only IHC 3+ as “HER2‐positive”, may be misleading. Such a label could implicitly assume a high stringency threshold (e.g., requiring ≥ 50% of cells with strong staining for mCRC according to HER2 amplification for colo‐rectaL cancer enhanced stratification criteria) or, conversely, ignore clinically relevant low‐level HER2 expression (IHC 1+ or 2+) that might still respond to ADC therapy. This oversimplification risks misclassification of HER2 status and suboptimal treatment decisions, especially in tissue‐agnostic HER2‐targeted treatment frameworks.

**Table 1:** Representative clinical trials of HER2‐targeted therapy.

Treatments	Registry no.	Tumor type; setting (*n*)	HER2 criteria	Efficacy outcomes
Carboplatin/paclitaxel ± trastuzumab (phase II) [57, 58]	NCT01367002	Uterine serous carcinoma (61)	IHC 3+ or IHC 2+/FISH+ (IHC 3+, intense complete, or basolateral/lateral membrane staining in > 30% tumor cells; IHC 2+, intense complete, or basolateral/lateral membrane staining in ≤ 30% tumor cells, or weak to moderate staining in ≥ 10% of tumor cells)	mPFS (8.0 months vs. 12.9 months), mOS (24.4 months vs. 29.6 months) in the carboplatin/paclitaxel group (*n* = 28), and the carboplatin/paclitaxel plus trastuzumab group (*n* = 30), respectively
Trastuzumab + lapatinib (phase II; HERACLES) [59]	EudraCT 2012‐002128‐33	Treatment‐refractory, KRAS codon 12/13 wild‐type metastatic colorectal cancer (27)	IHC 3+ or IHC 2+/FISH+ (tumors with intense membranous staining in ≥ 50% of cells by IHC or with 2 + IHC score and an HER2:CEP17 ratio ≥ 2 in more than 50% of cells by FISH)	ORR 30% (95% CI 14–50)
Tucatinib + trastuzumab (phase II) [60]	NCT03043313	Chemotherapy‐refractory, RAS wild‐type unresectable or metastatic CRC (84)	IHC 3+ or IHC 2+/ISH+ or NGS+ (determined at local laboratories certified by Clinical Laboratory Improvement Amendments or accredited by the International Organization for Standardization)	ORR 38.1% (95% CI 27.7–49.3)
T‐DXd 5.4 or 6.4 mg/kg Q3W (phase II; DESTINY‐Lung01 cohorts 1 and 1 A) [11]	NCT03505710	NSCLC; refractory to standard therapies (90)	IHC 2+ or 3+ (FDA approval for IHC 3+ solid tumors) (according to the 2016 ASCO/CAP GEA HER2 testing guidelines)	Cohort 1A (5.4 mg/kg, *n* = 41): ORR 34.1%, mDOR 6.2 months, mPFS 6.7 months, and mOS 11.2 months. Cohort 1 (6.4 mg/kg, *n* = 49): ORR 26.5%, mDOR 5.8 months, mPFS 5.7 months, and mOS 12.4 months
T‐DXd 5.4 mg/kg versus 6.4 mg/kg Q3W (phase II; DESTINY‐CRC02) [12]	NCT04744831	CRC; previously treated (122)	IHC 3+ or IHC 2+/ISH+ (FDA approval for IHC 3+ solid tumors) (according to the 2016 ASCO/CAP GEA HER2 testing guidelines)	ORR 37.8% versus 27.5%, mDOR 5.5 months versus 5.5 months, mPFS 5.8 months versus 5.5 months, mOS 13.4 months versus NE months in the 5.4 (*n* = 82) and 6.4 (*n* = 40) mg/kg arms, respectively
T‐DXd 5.4 mg/kg Q3W (phase II; DESTINY‐PanTumor02) [10]	NCT04482309	Solid tumors (267)	IHC 3+ or 2+ (FDA approval for IHC 3+ solid tumors) (according to the 2016 ASCO/CAP GEA HER2 testing guidelines)	IHC 3+ group (*n* = 75): ORR 61.3%, mDOR 22.1 months, mPFS 11.9 months, and mOS 21.1 months. IHC 2+ group (*n* = 125): ORR 27.2%, mDOR 9.8 months, mPFS 5.4 months, and mOS 12.2 months
Disitamab vedotin (phase II) [61]	NCT03507166	Urothelial carcinoma; previously treated (43)	IHC 2+ or 3+ (NMPA conditional approval) (according to the 2013 ASCO/CAP breast cancer HER2 testing guidelines)	ORR 51.2%, mDOR 6.9 months, mPFS 6.9 months, and mOS 13.9 months
Zanidatamab (HER2 ECD II and IV); phase II (HERIZON‐BTC‐01) [62]	NCT04466891	Biliary tract cancer, advanced‐stage after prior gemcitabine‐based chemotherapy (87)	IHC 2+ or 3+ (FDA approval for IHC 3+) (IHC 3+: Intense complete, basolateral, or lateral membranous reactivity in ≥ 10% of tumor cells)	Cohort 1 (HER2 IHC 3+ or 2+; *n* = 80): ORR 41.3%, mDOR 12.9 months, and mPFS 5.5 months. Cohort 2 (HER2 IHC 1+ or 0; *n* = 7): ORR 0% and mPFS 1.9 months

Abbreviations: ASCO, American Society of Clinical Oncology; CAP, College of American Pathologists; CEP17, chromosome 17 centromere; CRC, colorectal carcinoma; ECD, extracellular domain; FDA, Food and Drug Administration; FISH, fluorescence in situ hybridization; GEA, gastroesophageal adenocarcinoma; HER2, human epidermal growth factor receptor 2; HERACLES, HER2 amplification for colo‐rectaL cancer enhanced stratification; IHC, immunohistochemistry; ISH, in situ hybridization; KRAS, Kirsten rat sarcoma viral proto‐oncogene; mDOR, median duration of response; mOS, median overall survival; mPFS, median progression‐free survival; NGS, next‐generation sequencing; NMPA, National Medical Products Administration; NSCLC, non‐small‐cell lung cancer; ORR, objective response rate; OS, overall survival; PFS, progression‐free survival; T‐DXd, trastuzumab deruxtecan.

In light of these challenges, this consensus recommends aligning HER2 IHC scoring practices with regulatory‐approved clinical evidence and established guidelines. Instead of using a simplistic “positive” or “negative” classification, adopting the four‐tier scoring system (0, 1+, 2+, 3+) across different tumors is advised to standardize interpretation (Table 2) [13]. Furthermore, emerging evidence suggests that tumors with HER2 ultralow expression (IHC score below 1+) may still exhibit limited responsiveness to novel ADCs, such as T‐DXd and DV, due to bystander killing effects [63, 64]. Therefore, for cases with an HER2 score of 0, further differentiation is recommended. If there is a complete absence of staining, it can be reported as HER2 0 (no staining); otherwise, the percentage and intensity of HER2‐stained cells should also be reported. However, the reproducibility of ultralow detection is hindered by interobserver variability and assay sensitivity. Further standardization and validated antibody platforms are required before HER2 ultralow status can be routinely incorporated into clinical decision‐making.

**Table 2:** Scoring guidelines for the interpretation of HER2 IHC in pan‐tumor.

Surgical specimen	Biopsy specimen	Score
No reactivity or membranous reactivity in < 10% of tumor cells	No reactivity or no membranous reactivity in any tumor cell	0
Faint/barely perceptible membranous reactivity in ≥ 10% of tumor cells; cells are reactive only in part of their membrane	Tumor cell cluster with a faint/barely perceptible membranous reactivity, irrespective of the percentage of tumor cells stained	1+
Weak to moderate, complete, basolateral, or lateral membranous reactivity in ≥ 10% of tumor cells	Tumor cell cluster with a weak to moderate, complete, basolateral, or lateral membranous reactivity, irrespective of the percentage of tumor cells stained	2+
Strong, complete, basolateral, or lateral membranous reactivity in ≥ 10% of tumor cells	Tumor cell cluster with a strong, complete, basolateral, or lateral membranous reactivity, irrespective of the percentage of tumor cells stained	3+

*Note:* Tumor cell cluster (≥ 5 cohesive tumor cells).

Abbreviations: HER2, human epidermal growth factor receptor 2; IHC, immunohistochemistry.

**Figure 1:**
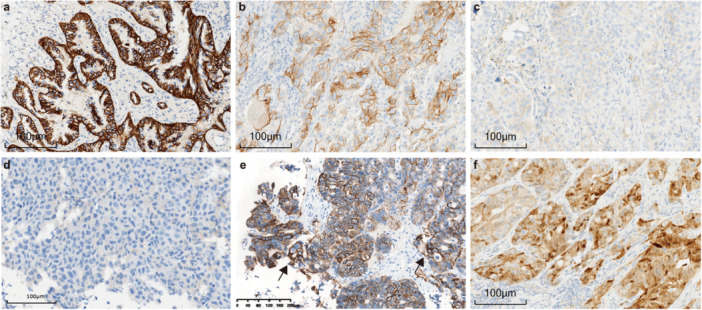
Representative images of HER2 immunohistochemistry staining. Figures (a)–(c) are from surgical specimens of lung adenocarcinoma. (a) Tumor cells show strong staining with either complete or basolateral membrane staining (×400). (b) The majority of tumor cells exhibit moderate staining (×400). (c) Tumor cells display weak staining (×400). (d) Biopsy specimen of lung adenocarcinoma showing complete absence of staining in tumor cells (×400). (e) Colon adenocarcinoma biopsy with most tumor cells showing moderate to weak staining, but clusters of strongly stained tumor cells are visible (arrow) (×100). (f) Lung adenocarcinoma resection specimen in which tumor cell cytoplasmic staining is observed and thus is not eligible for scoring (×400). All cases were stained with the HER2 (4B5) monoclonal antibody on the Ventana BenchMark Ultra platform using the Ultraview detection kit. HER2, human epidermal growth factor receptor 2.

During HER2 IHC interpretation, initial examination of the entire slide under low magnification should be performed to assess the staining quality and check for HER2 expression heterogeneity. Pathologists should avoid interpreting HER2 staining at tissue edges, in necrotic areas, or in regions with crush artifacts, and should focus only on well‐preserved invasive tumor regions. Critically, only membranous staining, whether complete, lateral, or basolateral, should be considered for HER2 scoring, with cytoplasmic staining disregarded. In addition, to improve reproducibility and reduce subjectivity, the “microscopic magnification rule” should be strictly adhered to: strong membranous staining should be discernible at low magnification (×2.5–×5), moderate staining at intermediate magnification (×10–×20), and weak staining only at high magnification (×40) (Figure 1) [65].

The heterogeneity of HER2 expression lacks a clear definition, and this issue becomes particularly complex in the pan‑tumor context. However, the core of the report should not be to document all heterogeneity, but to closely align with the companion diagnostic criteria of targeted therapies. When heterogeneity may influence clinical treatment eligibility or potentially affect therapeutic outcomes, it may be appropriate to include a relevant description, such as specifying the percentage of tumor cells at different staining intensities.

HER2 biology varies significantly by tissue origin—gene amplification dominates in breast and gastric cancers, while transcriptional upregulation or mutations occur in lung and bladder cancers [66]. These biological differences necessitate tumor‐type‐specific diagnostic algorithms, which may include integrated IHC, ISH, and NGS strategies. In clinical practice, while reflex ISH testing for IHC 2+ cases is generally recommended, it should not be mandated in the pathology report. Instead, the decision to perform confirmatory ISH should be made by the treating physician based on the clinical context. Specifically, for tumors such as NSCLC, discordant IHC 2+/3+, and ISH‐negative results should not be dismissed as assay artifacts. Instead, they often reflect nonamplified *HER2* activation. Therefore, for specified tumor types, laboratories may need to adapt HER2 evaluation frameworks to account for underlying oncogenic mechanisms while preserving scoring standardization.

*Recommendation* 4: In the interpretation of HER2 IHC results for pan‐tumor, it is recommended to use the four‐tier scoring system (0 to 3+) based on staining intensity and a threshold of 10% (surgical specimens) or 5 cohesive tumor cells (biopsy specimens) (evidence quality, Ⅱ; strength of recommendation, A).

*Recommendation* 5: HER2 IHC scoring of invasive cancer cells should be strictly performed according to HER2 testing guidelines and the “microscopic magnification rule.” When results are close to the threshold and scoring is difficult to determine, consultation with a second pathologist for a comprehensive evaluation is recommended (evidence quality, I; strength of recommendation, A).

### 5.6 Report Template

This template outlines the key elements necessary for reporting. To ensure accurate clinical interpretation, HER2 IHC reports must document the test results alongside the interpretation criteria employed. Furthermore, given the variability in antibodies and platforms, the specific testing procedure should be clearly described.

**Table:** 

**Reporting Template**
Testing Performed on Block Number(s) (specify): _________________
Specimen Type
___ Biopsy/curettage
___ Resection
___ Other (specify): _________________
Block Fixation and Processing
___ Formalin‐fixed, paraffin‐embedded
___ Other (specify): _________________
Appropriate Controls Verified
___ Yes
___ No
___ Other (specify): _________________
Test type
___ Administration cleared (specify test/vendor): ___________________
___ Laboratory‐developed test
Primary antibody
___ 4B5
___ HercepTest
___ Other (specify): ________________________________
HER2 IHC score (based on Pan‐tumor guidelines)
___ Score: □ 0
□ 1+
□ 2+ (Confirmatory ISH testing is recommended at clinical discretion.)
□ 3+

*Recommendation* 6: An HER2 IHC report should, at a minimum, include the testing platform and antibody used, as well as the test results and the interpretation criteria applied (evidence quality, Ⅱ; strength of recommendation, A).

### 5.7 Quality Control

IHC testing should incorporate external controls to ensure accuracy and reliability. Tumor tissue microarrays or cell lines exhibiting a range of HER2 staining intensities (from negative to strong) should be used as controls. This provides a more comprehensive approach to quality control, helping pathologists differentiate membranous staining intensities and validating the sensitivity and accuracy of IHC staining [14]. Routine analysis of positivity rates for different IHC scores is also encouraged to detect potential biases and support overarching quality control measures.

Laboratories performing HER2 testing must comply with national quality standards and obtain accreditation from relevant regulatory bodies [67]. These laboratories are required to participate in annual quality control programs, such as the Pathology Quality Control Center (PQCC), the National Cancer Center Proficiency Test Project, or other interlaboratory quality assessment initiatives [68]. Besides, testing personnel must possess the appropriate educational background, work experience, and professional training, along with proper certification. They must strictly adhere to established standard operating procedures to ensure the accuracy and integrity of HER2 testing.

*Recommendation* 7: A set of external controls with different staining gradients should be established. Additionally, laboratories must participate in regular quality control programs (evidence quality, I; strength of recommendation, A).

## 6 Conclusions

HER2 overexpression testing has emerged as a critical component in the management of various malignancies, particularly in the context of pan‐tumor applications driven by advancements in molecular profiling and targeted therapy. While HER2‐targeted therapies have demonstrated promising efficacy across a range of HER2‐positive solid tumors, variability in testing methodologies, scoring criteria, and reporting practices continues to pose challenges to the consistent application of these treatments. Recognizing the growing importance of HER2 as a predictive biomarker, a multidisciplinary panel of experts has developed a consensus framework aimed at standardizing HER2 testing across diverse tumor types.

The recommendations advocate for the use of validated testing platforms, adherence to standardized protocols for specimen processing, and the adoption of a four‐tier scoring system (0 to 3+) to ensure reproducibility and accuracy. Besides, robust quality control measures and external controls are essential to maintain diagnostic reliability. The consensus further emphasizes the need for laboratory accreditation and periodic participation in quality assurance programs to uphold standards across clinical settings.

Given the heterogeneity of HER2 expression across tumor types and the evolving landscape of molecular oncology, this unified approach aims to optimize patient selection for HER2‐targeted therapies and improve therapeutic outcomes. As novel diagnostic tools and treatment paradigms continue to emerge, future updates to these guidelines will be critical to ensure their alignment with the latest scientific and clinical advancements. By establishing a standardized framework for pan‐tumor HER2 testing, this consensus aims to bridge existing gaps, facilitating broader access to effective HER2‐targeted therapies and advancing precision oncology for patients with HER2‐expressing malignancies.

## Author Contributions

**Zhe Wang:** conceptualization (lead), writing – original draft (lead). **Lin Chen:** conceptualization (lead), writing – original draft (lead). **Wei Rao:** conceptualization (lead), writing – original draft (lead). **Baoshan Cao:** writing – review and editing (supporting). **Muyan Cai:** writing – review and editing (supporting). **Xiangshan Fan:** writing – review and editing (supporting). **Yuan Ji:** writing – review and editing (supporting). **Yuan Li:** writing – review and editing (supporting). **Yan Song:** writing – review and editing (supporting). **Yan Sun:** writing – review and editing (supporting). **Yu Sun:** writing – review and editing (supporting). **Chunyan Wu:** writing – review and editing (supporting). **Qingxin Xia:** writing – review and editing (supporting). **Yanfeng Xi:** writing – review and editing (supporting). **Liyan Xue:** writing – review and editing (supporting). **Rutie Yin:** writing – review and editing (supporting). **Han Liang:** conceptualization (lead), project administration (lead), supervision (lead), writing – review and editing (lead). **Jianming Ying:** conceptualization (lead), writing – review and editing (lead), project administration (lead), supervision (lead).

## Funding

The study was supported by National High Level Hospital Clinical Research Funding.

## Ethics Statement

The authors have nothing to report.

## Consent

The authors have nothing to report.

## Conflicts of Interest

The authors declare no conflicts of interest.

## Supporting information

Supporting File

## Data Availability

The authors have nothing to report.
