# Supplementary material for: Consensus on Human Epidermal Growth Factor Receptor 2 Overexpression Testing in Pan‐Tumor
Source: Cancer Innov. 2026 Apr 21;5(2):e70060. doi: 10.1002/cai2.70060 (PMC13098049; doi:10.1002/cai2.70060)
Supplement: Supplementary file 1 — Supporting File [file CAI2-5-e70060-s001.docx]

**Supplemental Table 1** Quality of evidence and grading of expert recommendations.

| **Category, grade** | **Definition** |
| --- | --- |
| Strength of recommendation | |
| A | Good evidence to support a recommendation |
| B | Moderate evidence to support a recommendation |
| C | Poor evidence to support a recommendation |
| Quality of evidence | |
| I | Evidence from ≥ 1 properly randomized, controlled trial |
| II | Evidence from ≥ 1 well-designed clinical trial, without randomization; from cohort or case-control analytic studies (preferably from > 1 center); from multiple time series; or from dramatic results from uncontrolled experiments |
| III | Evidence from opinions of respected authorities, based on clinical experience, descriptive studies, or reports of expert committees |
